# Abnormal liver function induced by myofibroblastic sarcoma infiltrating the liver: A case report

**DOI:** 10.3892/ol.2014.2740

**Published:** 2014-11-27

**Authors:** YE PAN, XIAOLU WU, JIAJUN LIU, AIKEREMUJIANG MUHEREMU

**Affiliations:** 1 Department of Infectious Diseases, The First Affiliated Hospital of Xiamen University, Xiamen 361003, P.R. China; 2 Department of Orthopaedic Oncology Surgery, Beijing Ji Shui Tan Hospital, Beijing 100035, P.R. China

**Keywords:** myofibroblastic sarcoma, liver, case report

## Abstract

Myofibroblastic sarcoma (MS) is a rare disease, which most frequently occurs in the head, neck and extremities. To the best of our knowledge, although a small number of studies have reported MS of the oral cavity, maxilla, tonsil, thyroid and tongue, MS of the liver and subsequent disabled liver function has not been previously reported. This study presents the case of a 38 year old female initially diagnosed and treated for a mass in the retroperitoneal region, who was subsequently diagnosed with MS of the liver three months following preliminary surgical treatment. The patient refused further treatment and was lost to follow-up three months after discharge from the hospital. Clinical, biochemical and imaging observations, as well as pathological manifestations of the patient in the present case are discussed with the aim of increasing the knowledge of MS of the liver.

## Introduction

Myofibroblasts are a type of interstitial spindle cell with the ultrastructural characteristics of both fibroblasts and smooth muscle cells. Myofibroblasts were first identified electromagnetically by Gabbiani *et al* (1) in 1971. Montgomery *et al* (2) initially reported myofibroblastic sarcoma (MS) in 2001. MS originates from undifferentiated mesenchymal cells with the potential to differentiate into fibrocytes and histiocytes. According to the classification of the World Health Organization 2002, myofibrosarcoma was defined as low-grade MS (LGMS) (3). LGMS is a neoplasm of atypical myofibroblasts with fibromatoses like features and a predilection for head and neck sites (4). LGMS is rare in clinical practice and can occur at any site. The head and neck, extremities and femoral bone are the most common sites of origin (5–8), whereas occurrence in the abdominal cavity is extremely rare and thus, only a small number of cases have been reported in the literature (9–14).

To the best of our knowledge, no cases of MS of the liver have been reported. The current study presents the pathological observations, as well as the clinical manifestations, biochemical blood test results and imaging results of a patient with MS in the right posterior liver. Written informed consent for the publication of this study was obtained from the patient.

## Case report

**Figure 1:**
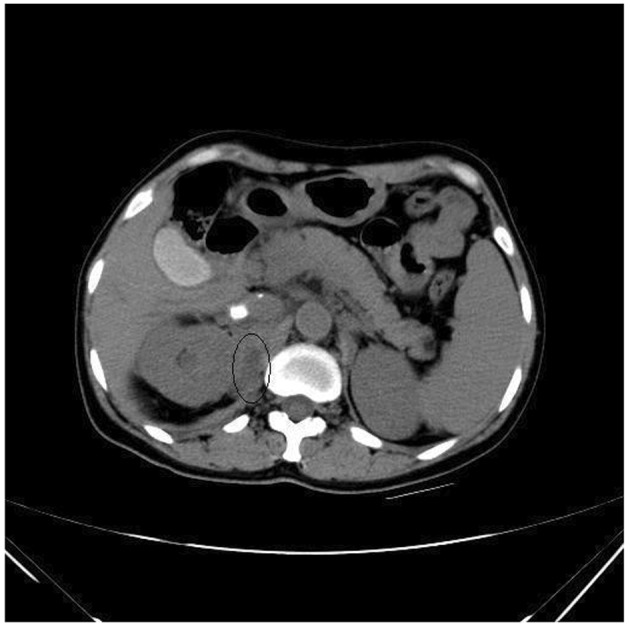
Computed tomography scan revealing a mass in the retroperitoneal region.

**Figure 2:**
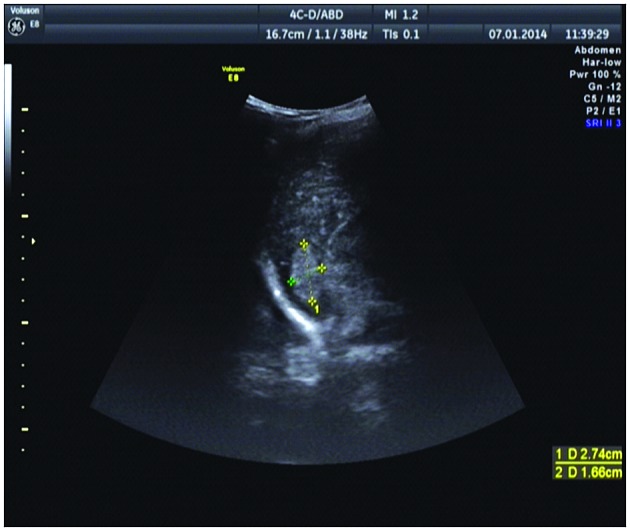
B-ultrasonic examination revealing a mass in the retroperitoneal region.

**Figure 3:**
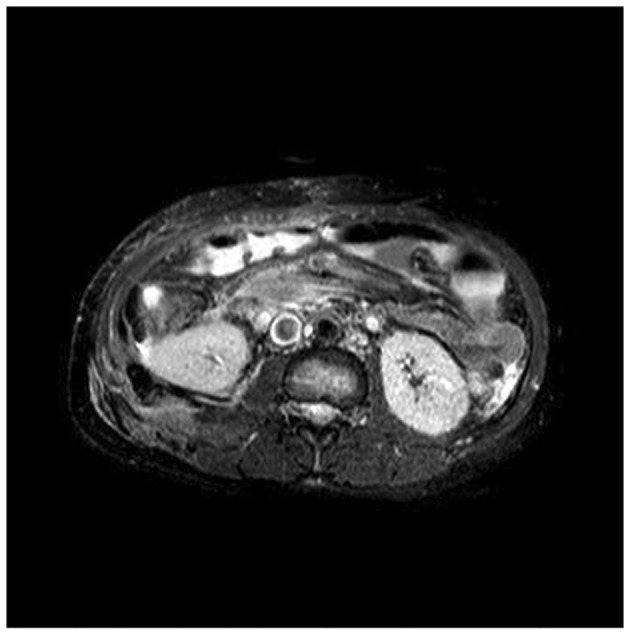
Magnetic resonance imaging showing postsurgical examination of the abdomen.

A 38 year old female presented to the Clinic of General Surgery, Department of Infectious Diseases, The First Affiliated Hospital of Xiamen University (Xiamen, China), after experiencing backache for three months previously. The patient was diagnosed with a retroperitoneal mass following computed tomography (CT) and ultrasonic tests (Figs. 1 and 2), which was removed surgically. Postoperative magnetic resonance imaging (Fig. 3) and pathology revealed an inflammatory myofibroblastic tumor, borderline type, according to the World Health Organization Classification of Tumors (3).

Following surgery, the patient was treated with reduced glutathione (300 mg/day) and phosphatidylcholine (200 mg/day) for a month. A liver function test one month after surgery revealed total bilirubin (TBIL) levels of 13.8 μmol/l (normal range, 0–20.5 μmol/l), alanine aminotransferase (ALT) levels of 78 μ/l (normal range, 0–45 U/l), aspartate aminotransferase (AST) levels of 105 μ/l (normal range, 0–35 U/l), alkaline phosphatase (ALP) levels of 458 μ/l (normal range, 40–150 U/l), and gamma glutamyltransferase (GGT) levels of 225 μ/l (normal range, 0–50 U/l). The patient exhibited no symptoms of fever, jaundice, nausea, loss of appetite, fatigue or diarrhea, however, no significant improvement in liver function was observed when compared with that prior to surgery. Three months following surgery, the patient’s liver function was evaluated, which revealed TBIL levels of 16.1 μmol/l, ALT levels of 165 μ/l, AST levels of 127 μ/l, GGT levels of 438 μ/l and ALP levels of 1426 μ/l. The patient was transferred from surgical clinic to our department (the Department of Infectious Diseases, The First Affiliated Hospital of Xiamen University), and was admitted with a diagnosis of liver damage of unknown origin.

**Figure 4:**
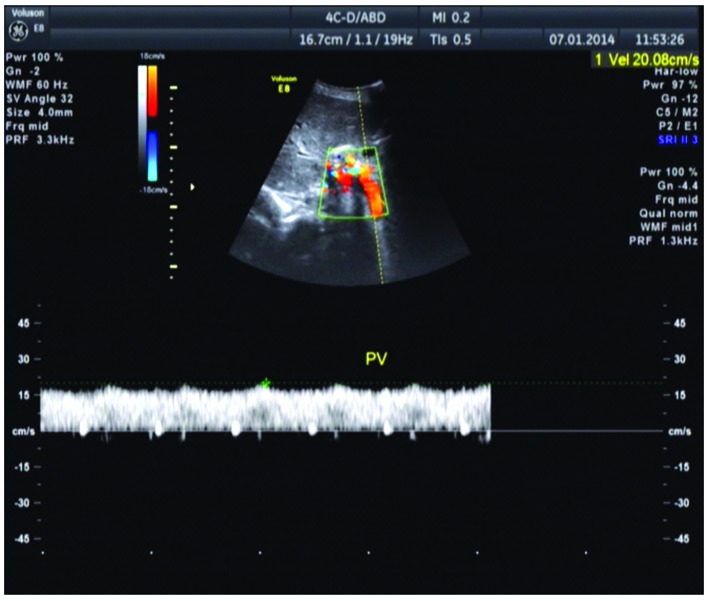
Color ultrasound of the upper abdomen showing diffuse disease of the hepatic parenchyma.

**Figure 5:**
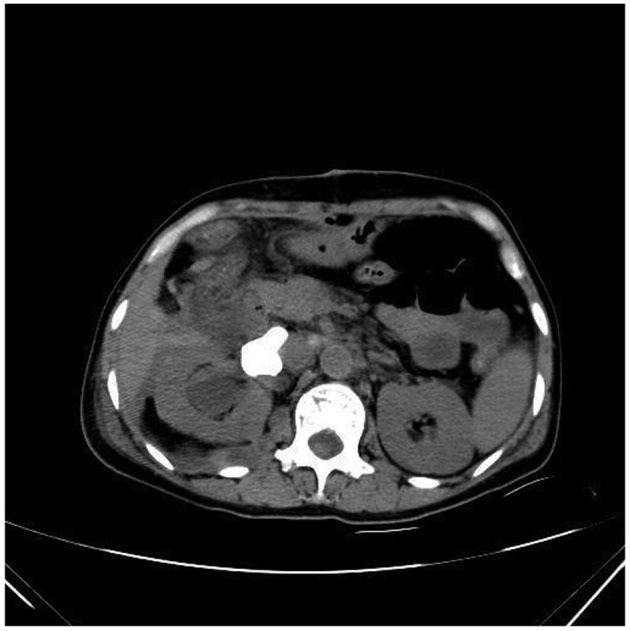
Plain and enhanced computed tomography scan of the liver showing a slight reduction in volume and cavernous hemangioma in the right posterior liver.

**Figure 6:**
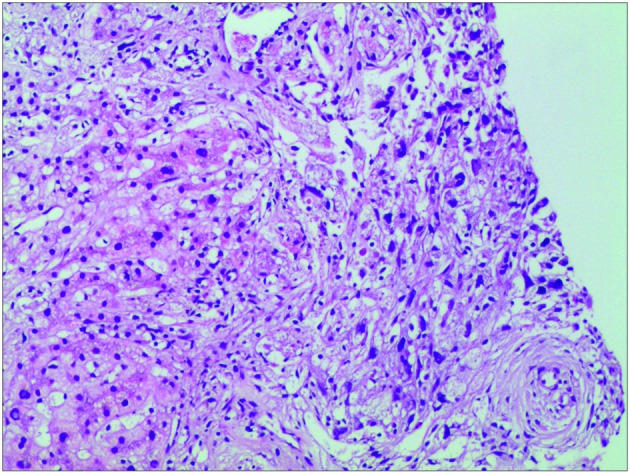
Infiltration of spindle type tumor cells in the liver tissue. (Hematoxylin and eosin staining; magnification, ×100).

Physical examination on admission to our department revealed that the patient showed no mental or intellectual abnormalities, with no jaundice of the skin or sclera and no liver palm or spider angiomas. Lung sounds were clear, with no dry or moist rales. The patient’s heart rhythm was regular and no murmurs were heard. The abdomen was flat and soft and a diagonal surgical incision 20 cm in length was identified on the abdomen. No tenderness or rebound tenderness was identified. The liver was palpable under the inferior margin of the rib. However, xiphoid bone and the spleen were not palpable under the left rib. The patient was negative for Murphy syndrome and no shifting dullness of the abdomen or swelling of the lower extremities was identified. Levels of α-fetoprotein (AFP) were <1.0 ng/ml (normal range, 0–9 ng/ml). Levels of AFP glycosylation heterogeneity were normal (<10%). Tests for viral markers of hepatitis A, B, C, D and E were negative. Furthermore, antibody profiles of autoimmune liver disease and auto-antibodies were negative: IgG, 22.200 g/l; IgA, 3.8200 g/l; IgM, 2.4200 g/l; and IgG2, 11.0 g/l. Color ultrasound of the upper abdomen showed diffuse disease of the hepatic parenchyma (Fig. 4). Plain and enhanced CT scans of the liver revealed a marginal reduction in volume and cavernous hemangioma in the right posterior liver (Fig. 5). Liver biopsy was performed to clarify the nature of the lesion (Fig. 6). Pathological observation revealed widespread infiltration of spindle-shaped cells, with abundant and eosinophilic cytoplasm, unclear cell borders and low and moderate range atypia of the nuclei. A small number of lymphocytes and eosinophilic granulocytes were also observed in the liver tissue. In the surrounding liver tissue lesions, the dividing lines of liver lobules were clear, and edema and spotted and focal necrosis was present in a small number of liver cells. The portal area had marginally expanded and the infiltration of lymphocytes and eosinophilic granulocytes was identified. Immunohistochemical analysis revealed positivity for smooth muscle actin, CD99, Bcl-2, P53, calponin and vimentin and negativity for cytokeratin (CK), CK8, glypican-3, Hep, myoglobin, myosin, desmin, S100, HMB45, CD117, CD34 and anaplastic lymphoma kinase. MASSON + reticular fiber staining revealed a mesh stent in the hepatic lobule, with a clear structure and slight hyperplasia of fibrous tissue in the portal area. Pathological diagnosis indicated infiltration of low grade MS in the liver. After diagnosis was confirmed, the patient was transferred to the Department of Surgical Oncology, The First Affiliated Hospital of Xiamen University for further treatment. The patient refused further treatment and was lost to follow-up three months after discharge from the hospital.

## Discussion

Myofibroblasts are a type of interstitial spindle cell with the ultrastructural characteristics of both fibroblasts and smooth muscle cells. They occur in a number of normal tissues and certain benign lesions, including infection and granulation tissue (15). In the current case, the pathology of the retroperitoneal mass indicated inflammatory myofibroblastic tumor (IMT), which is a tumor of intermediate type. No recurrence of the retroperitoneal mass was identified in the follow-up. Furthermore, no tumors were detected in the intra-abdominal organs, and the retroperitoneal IMT was a different type of tumor to LGMS. The patient initially presented with abnormal liver function, which was characterized by a marginal increase in ALT and AST levels and a significant increase in ALP, GGT and ALP levels.

In this case report, the female patient exhibited no evidence of viral hepatitis or liver disease induced by alcohol or drugs. A significant increase in ALP and GGT levels may lead to misdiagnosis of autoimmune liver disease. Liver iconography of the patient in the present report revealed no definite signs of space occupying lesions. Infiltration of tumor cells is easily detectable in routine liver biopsy, which may reveal widespread infiltration of tumor cells in the whole liver. However, at present, there are few reports of this type of liver lesion in the literature.

Further studies are required to investigate the bionomics of LGMS due to its rarity in clinical practice. It remains unclear whether the abnormality of liver function was caused by the tumor cells directly or by hepatic tissue destroyed by tumor cells. Therefore, the association between IMT and LGMS and tumorigenicity requires further study (16).
